# Epidemiology of the Rhinovirus (RV) in African and Southeast Asian Children: A Case-Control Pneumonia Etiology Study

**DOI:** 10.3390/v13071249

**Published:** 2021-06-27

**Authors:** Vicky L. Baillie, David P. Moore, Azwifarwi Mathunjwa, Henry C. Baggett, Abdullah Brooks, Daniel R. Feikin, Laura L. Hammitt, Stephen R. C. Howie, Maria Deloria Knoll, Karen L. Kotloff, Orin S. Levine, Katherine L. O’Brien, Anthony G. Scott, Donald M. Thea, Martin Antonio, Juliet O. Awori, Amanda J. Driscoll, Nicholas S. S. Fancourt, Melissa M. Higdon, Ruth A. Karron, Susan C. Morpeth, Justin M. Mulindwa, David R. Murdoch, Daniel E. Park, Christine Prosperi, Mohammed Ziaur Rahman, Mustafizur Rahman, Rasheed A. Salaudeen, Pongpun Sawatwong, Somwe Wa Somwe, Samba O. Sow, Milagritos D. Tapia, Eric A. F. Simões, Shabir A. Madhi

**Affiliations:** 1 South African Medical Research Council Vaccines and Infectious Diseases Analytics Research Unit, Faculty of Health Sciences, University of the Witwatersrand, Johannesburg 2050, South Africa; David.Moore@wits.ac.za (D.P.M.); azwifarwim@nicd.ac.za (A.M.); Eric.Simoes@ucdenver.edu (E.A.F.S.); madhis@rmpru.co.za (S.A.M.); 2 Department of Science and Technology/National Research Foundation: Vaccine Preventable Diseases Unit, University of the Witwatersrand, Johannesburg 1864, South Africa; 3 Department of Paediatrics & Child Health, Chris Hani Baragwanath Academic Hospital and University of the Witwatersrand, Johannesburg 1864, South Africa; 4 Division of Global Health Protection, Thailand Ministry of Public Health–U.S. Centers for Disease Control and Prevention Collaboration, Nonthaburi 11000, Thailand; hfb8@cdc.gov (H.C.B.); hps5@cdc.gov (P.S.); 5 Division of Global Health Protection, Center for Global Health, Centers for Disease Control and Prevention, Atlanta, GA 30333, USA; 6 Department of International Health, Johns Hopkins Bloomberg School of Public Health, Baltimore, MD 21205, USA; wbrooks3@jhu.edu; 7 International Centre for Diarrhoeal Disease Research, Bangladesh (icddr,b), Dhaka and Matlab, Bangladesh; mzrahman@icddrb.org (M.Z.R.); mustafizur@icddrb.org (M.R.); 8 Department of International Health, International Vaccine Access Center, Johns Hopkins Bloomberg School of Public Health, Baltimore, MD 21205, USA; drf3217@gmail.com (D.R.F.); lhammitt@jhu.edu (L.L.H.); mknoll2@jhu.edu (M.D.K.); orin.levine@gatesfoundation.org (O.S.L.); obrienk@who.int (K.L.O.); adriscoll@som.umaryland.edu (A.J.D.); nick.fancourt@menzies.edu.au (N.S.S.F.); mhigdon@jhu.edu (M.M.H.); danpark@email.gwu.edu (D.E.P.); cprospe1@jhu.edu (C.P.); 9 Division of Viral Diseases, National Center for Immunizations and Respiratory Diseases, Centers for Disease Control and Prevention, Atlanta, GA 30333, USA; 10 Kenya Medical Research Institute-Wellcome Trust Research Programme, Kilifi 80108, Kenya; anthony.scott@lshtm.ac.uk (A.G.S.); JWOtieno@kemri-wellcome.org (J.O.A.); susan.morpeth@middlemore.co.nz (S.C.M.); 11 Medical Research Council Unit at the London School of Hygiene and Tropical Medicine, Basse 273, The Gambia; stephen.howie@auckland.ac.nz (S.R.C.H.); Martin.Antonio@lshtm.ac.uk (M.A.); rsalaudeen@mrc.gm (R.A.S.); 12 Department of Paediatrics: Child & Youth Health, University of Auckland, Park Rd, Auckland 1023, New Zealand; 13 Division of Infectious Disease and Tropical Pediatrics, Department of Pediatrics, Center for Vaccine Development and Global Health, University of Maryland School of Medicine, Baltimore, MD 21205, USA; kkotloff@som.umaryland.edu (K.L.K.); MTAPIA@som.umaryland.edu (M.D.T.); 14 Department of Infectious Disease Epidemiology, London School of Hygiene & Tropical Medicine, London WC1E 7HT, UK; 15 Department of Global Health, Boston University School of Public Health, Boston, MA 02118, USA; dthea@bu.edu; 16 Department of Pathogen Molecular Biology, London School of Hygiene & Tropical Medicine, Microbiology and Infection Unit, Warwick Medical School, University of Warwick, Coventry CV4 7JJ, UK; 17 Center for Vaccine Development and Global Health, University of Maryland School of Medicine, Baltimore, MD 21205, USA; 18 Department of International Health, Center for Immunization Research, Johns Hopkins Bloomberg School of Public Health, Baltimore, MD 21205, USA; rkarron@jhu.edu; 19 Microbiology Laboratory, Middlemore Hospital, Counties Manukau District Health Board, Auckland 1640, New Zealand; 20 Department of Paediatrics and Child Health, University Teaching Hospital, Lusaka 50110, Zambia; jmm7503@yahoo.co.uk (J.M.M.); ssow@som.umaryland.edu (S.W.S.); 21 Department of Pathology and Biomedical Sciences, University of Otago, Christchurch 8011, New Zealand; david.murdoch@otago.ac.nz; 22 Microbiology Unit, Canterbury Health Laboratories, Christchurch 8140, New Zealand; 23 Milken Institute School of Public Health, Department of Epidemiology, George Washington University, Washington, DC 20052, USA; 24 Medical Microbiology Department, Lagos University Teaching Hospital, Lagos 100254, Nigeria; 25 Centre pour le Développement des Vaccins (CVD-Mali), Bamako 198, Mali; somwewa@yahoo.com; 26 Department of Pediatrics, University of Colorado School of Medicine and Center for Global Health, Colorado School of Public Health, Aurora, CO 80309, USA

**Keywords:** rhinovirus, epidemiology, childhood, pneumonia, PERCH

## Abstract

Rhinovirus (RV) is commonly detected in asymptomatic children; hence, its pathogenicity during childhood pneumonia remains controversial. We evaluated RV epidemiology in HIV-uninfected children hospitalized with clinical pneumonia and among community controls. PERCH was a case-control study that enrolled children (1–59 months) hospitalized with severe and very severe pneumonia per World Health Organization clinical criteria and age-frequency-matched community controls in seven countries. Nasopharyngeal/oropharyngeal swabs were collected for all participants, combined, and tested for RV and 18 other respiratory viruses using the Fast Track multiplex real-time PCR assay. RV detection was more common among cases (24%) than controls (21%) (aOR = 1.5, 95%CI:1.3–1.6). This association was driven by the children aged 12–59 months, where 28% of cases vs. 18% of controls were RV-positive (aOR = 2.1, 95%CI:1.8–2.5). Wheezing was 1.8-fold (aOR 95%CI:1.4–2.2) more prevalent among pneumonia cases who were RV-positive vs. RV-negative. Of the RV-positive cases, 13% had a higher probability (>75%) that RV was the cause of their pneumonia based on the PERCH integrated etiology analysis; 99% of these cases occurred in children over 12 months in Bangladesh. RV was commonly identified in both cases and controls and was significantly associated with severe pneumonia status among children over 12 months of age, particularly those in Bangladesh. RV-positive pneumonia was associated with wheezing.

## 1. Introduction

Rhinovirus (RV) was first discovered in 1956 in individuals with mild respiratory tract infection [1]. However, since the advance in molecular diagnostic tools—namely, the polymerase chain reaction (PCR)—HRV has been recognized as one of the most prevalent respiratory viruses in children requiring hospitalization. RV has also been commonly detected in children with otitis media, sinusitis, asthma exacerbation, cystic fibrosis, bronchitis, and lower respiratory tract infection (LRTI) [2,3,4,5,6,7,8,9,10,11,12]. The role of RV in severe childhood LRTI, however, remains controversial. RV identification is ubiquitous in healthy, asymptomatic children [13], and virus shedding can persist for 10–14 days [14].

Several studies have examined RV prevalence in hospitalized children and healthy controls to determine the clinical significance of RV detection in both diseased and healthy individuals. In the majority of these studies, cases had a significantly higher prevalence of RV detected than controls [15,16,17,18,19]. However, these studies were not specifically designed to address the clinical epidemiology of RV and viral or bacterial coinfections in relation to disease severity [15,17,20]. Understanding the importance of HRV infection is critical when it comes to determining future strategies for disease treatment and prevention.

The Pneumonia Etiology for Child Health (PERCH) study has previously reported on the overall and high-level causes of pneumonia in children [21]. In this study, we specifically focus on the clinical epidemiology of RV infection, overall and by site, and its interactions with other respiratory pathogens in children 1–59 months of age hospitalized with pneumonia and in community controls.

## 2. Materials and Methods

### 2.1. Case and Control Definitions

**Figure 1:**
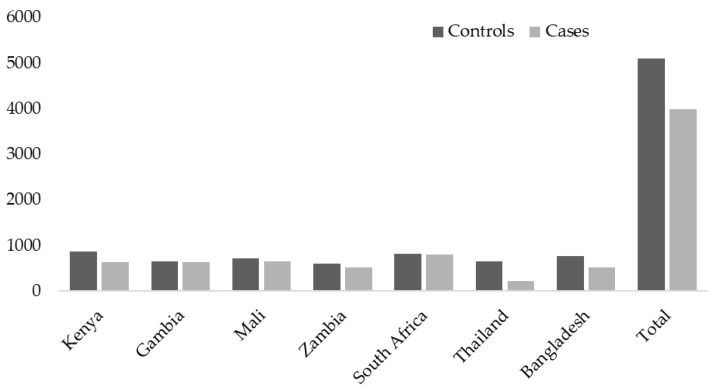
The number of cases and controls enrolled per country and overall.

The PERCH study was undertaken in seven countries, including South Africa, Mali, Zambia, Kenya, The Gambia, Bangladesh, and Thailand from August 2011 through January 2014 (Figure 1).

Details on enrollment of cases and controls, sample testing, and clinical evaluation in the PERCH study have been described elsewhere [21,22]. Briefly, pneumonia cases were children aged 28 days to 59 months hospitalized with World Health Organization (WHO)-defined severe or very severe pneumonia (according to the pre-2013 definitions) [23,24]. Controls were enrolled from the same communities as cases and included children without symptoms of severe or very severe pneumonia who were frequency-matched by age group and month of enrollment to the cases. For analysis, the controls were stratified into those with acute respiratory infections (ARI), defined as having (1) cough or runny nose or (2) ear discharge, wheezing or difficulty breathing, together with either a fever (temperature greater or equal to 38 °C in the past 48 h) or a sore throat, and those who were asymptomatic at the time of sampling (non-ARI).

### 2.2. Specimen Collection and Laboratory Testing

Flocked nasopharyngeal (NP) swabs (Flexible minitip, Copan ^®^, Murrieta, CA, USA) and rayon oropharyngeal (OP) swab specimens were collected from cases and controls upon enrollment. The swabs were combined in a single 3 mL of Universal Transport Media (Copan ^®^, CA, USA)-containing vial and kept at 4–8 °C for a maximum of 24 h, then archived at −70 °C until tested. Total nucleic acids were extracted from the combined NP/OP swabs using the NucleiSens EasyMag extraction system as per manufacturer’s instructions (BioMerieux, Marcy l’Etoile, France) and were tested in-country by multiplex PCR for evidence of 33 pathogens (FTD Resp 33, Fast-track Diagnostics, Sliema, Malta). Standard curves were used to calculate pathogen load from PCR cycle threshold values [25].

Other investigations included blood culture on cases using the BACTEC (Becton Dickinson, Sparks, MD, USA) in South Africa, Kenya, Mali, Zambia, and The Gambia. Thailand and Bangladesh used the BacT/Alert microbial system for blood culture (Organon Teknika, Durham, NC, USA). Induced sputum, and pleural fluid where clinically indicated, gastric aspirate, and lung aspirate (eligible cases only in The Gambia, Mali, South Africa, and Bangladesh) samples were collected and cultured using standard culture and biochemical tests. The induced sputum and pleural fluids were also tested using the FTD-33 respiratory panels. The FTD-33 RV results for the induced sputum showed substantial kappa concordance with the RV NP/OP results (0.60; *p* < 0.001). Further, the NP/OP specimens had the analytical advantage of being available for both the cases and the controls. Thus, only the NP/OP results were included in this and the previously published PERCH etiology analysis [26]. Pleural fluid specimens were also tested for pneumococcal antigen with the BinaxNow^®^ antigen detection kit (Alere, Orlando, Florida). Microbiologically confirmed pneumococcal pneumonia (MCPP) was defined as *Streptococcus pneumoniae* cultured from a normally sterile fluid. In addition, a case was considered to have MCPP if the pleural fluid or lung aspirate was FTD-33 PCR positive for pneumococcus or pneumococcus antigen positive on the BinaxNow^®^ assay.

### 2.3. Statistical Analysis

The analysis was limited to HIV-uninfected children, with the epidemiology of RV in relation to HIV infection status to be reported separately. PCR quantifications were log10 transformed. Chi-squared and Wilcoxon tests were used to analyze the demographic characteristics of cases and controls. Binary and multinomial logistic regression analyses were used to model the prevalence of RV within the study population. Age categories and site of enrolment, together with variables with an association at *p* < 0.2 in the univariate analysis, were included in the multivariable models. Reverse cumulative plots were used to analyze the relationship between RV NP/OP viral loads among cases compared with controls and whether RV viral load was associated with severity of disease among cases. All statistical analysis and reverse cumulative plots were performed using STATA Version 12.1 (College Station, TX, USA), and a two-sided *p*-value < 0.05 was considered statistically significant. Further, the PERCH integrated analysis (PIA) method, described in detail elsewhere [26,27,28], was used to estimate the percentage of pneumonia attributable to each pathogen, including RV. The PIA gave an estimate for both the individual- and population-level etiology probability distribution for each pathogen ranging from 0% to 100%, with >75% considered to be a higher probability of RV being the cause of pneumonia. The PIA assigned the probability of a pathogen being the cause of pneumonia based on the laboratory testing results and prior probability with 95% CI at both the population and individual level [26].

## 3. Results

### 3.1. Characteristics of Community Controls by RV Status

RV was detected in 21% of community controls and was more likely to be detected in controls with ARI (25%) than non-ARI children (20%; aOR = 1.6, 95%CI: 1.3–1.8), regardless of age group. This association was mainly driven by the Asian sites and Kenya (Supplementary Materials S1 and S2). We found no difference in NP/OP viral load between ARI controls and non-ARI controls among RV-positive participants. RV-positive controls were younger (mean age of 13.2 months) and more likely to have been born prematurely (gestational age <37 weeks) than those without RV infection (mean age of 16.1 months). The RV-positive controls were also more likely to have a respiratory tract infection with symptoms of rhinorrhea or cough compared with the RV-negative controls (Table 1). Thirty-eight percent of RV-positive controls were coinfected with other viruses compared with forty-seven percent of RV-negative controls. Common RV-positive co-infections included bocavirus (HBoV), adenovirus (AdV), and coronaviruses (HCoV), which were also identified with similar frequency among RV-negative controls (Table 1). There was also a similar prevalence in the detection of respiratory syncytial virus (RSV), human metapneumovirus (HMPV), and parainfluenza viruses (PIV) between RV-positive and RV-negative controls, while the prevalence of co-infection with influenza virus was lower among RV-positive controls (Table 1). RV-positive controls were more likely to be co-infected with common nasopharyngeal colonizing bacteria, including *M. catarrhalis, H. influenza*, and *S. pneumoniae*, compared with the RV-negative controls.

Among the RV-positive controls, those in whom RV was the only respiratory virus detected in the NP/OP swabs were younger than the children with RV together with other co-infecting viruses (Table 2). There were no other differences in demographics and in health and clinical characteristics between controls where RV was the only respiratory virus detected and RV-mixed viral infections (Table 2). The controls where RV was the only respiratory virus detected were, however, more likely to be co-infected with *S. pneumoniae* in the nasopharynx/oropharynx. Further, there were no differences in the RV viral load between these two groups (3.5 vs. 3.4 log10 copies/mL; *p* = 0.28).

### 3.2. RV infection Among Pneumonia Cases

Of the 3870 pneumonia cases, 68% were categorized as severe and 32% as very severe. RV was detected in 24% of cases, with no difference between severe and very severe cases. Although RV-positive very severe cases had higher NP/OP RV viral load (3.8 log10 copies/mL) than severe cases (3.6 log10 copies/mL, *p* = 0.01), we did not identify a threshold to discriminate disease severity using reverse cumulative plot or Youden indices (Supplementary Materials S1).

RV-positive cases were significantly older than those without RV infection (mean 13.1 vs. 11.2 months), but they were similar in other demographic characteristics (Table 3). Compared with RV-negative cases, RV-positive cases were more likely to have wheezing and tachypnea. Conversely, RV-positive cases were less likely to have radiographically confirmed pneumonia (chest X-ray with any infiltrate), to present with convulsions, and to have prolonged hospital stays (>3 days). There was no association between RV infection and the presence of hypoxia, mechanical ventilation, or case fatality. RV-positive cases were less likely than RV-negative cases to present with fever, alveolar consolidation on chest X-ray, and medically significant C-reactive protein levels (CRP ≥40 mg/L). RV-positive cases were, however, more likely to have leukocytosis. RV-positive compared with RV-negative cases were less likely to be infected with RSV, influenza virus, HMPV, and PIV. The individual site evaluation is available in Supplementary Materials S3 and S4, with similar trends as observed for the overall site comparisons.

Co-infection with at least one other respiratory virus was more common among RV-positive cases compared with RV-negative cases (Table 4). RV-positive cases without detection of other viruses (mono-RV) were older than those with co-infections, and in the multivariable analysis, adjusting for co-infecting bacteria, mono-RV cases had a higher case fatality ratio (7%) than those with co-infections (3%, aOR = 2.6, aOR 95%CI:1.2–5.5). There were other differences in demographics, clinical features, or markers of bacterial co-infections; however, the mono-RV cases were less likely to be co-infected with H. influenzae and M. catarrhalis in the nasopharynx/oropharynx. There were no differences in RV viral load between the mono-RV infected cases (3.8 log10 copies/mL) compared with the cases with mixed RV-viral infections (3.6 log10 copies/mL; *p* = 0.080).

**Figure 2:**
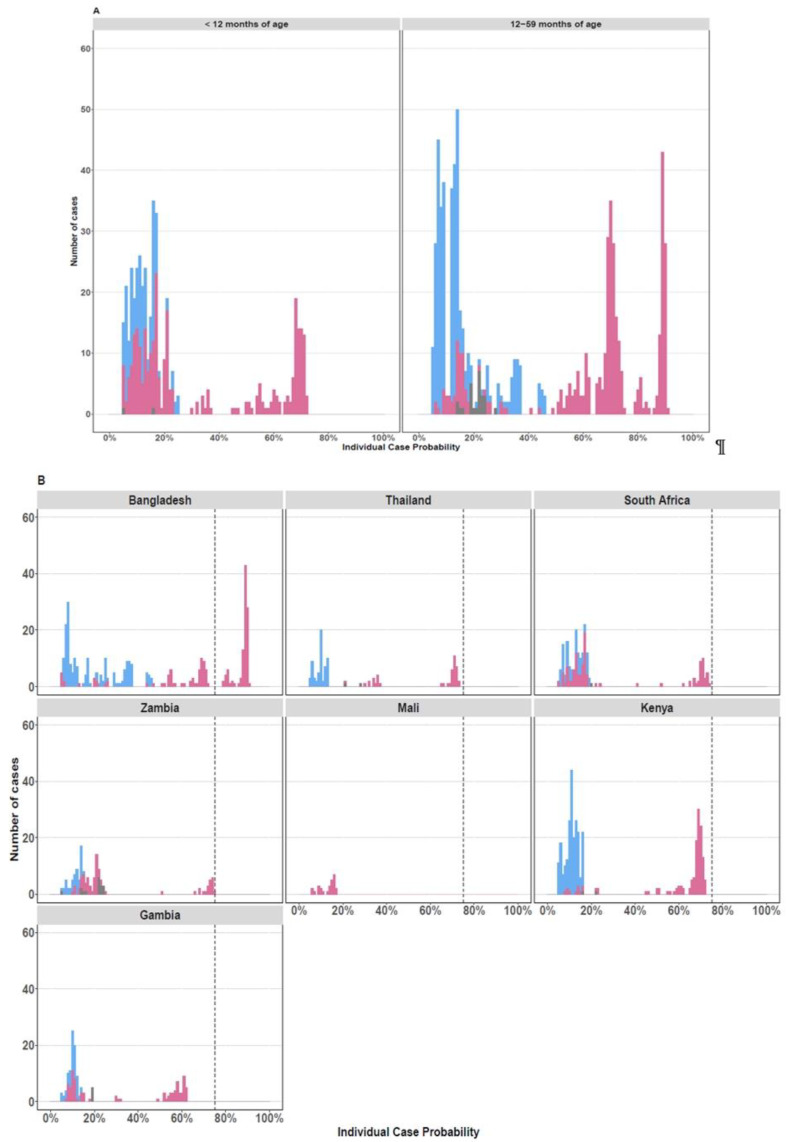
Individual Case Etiological Probability of rhinovirus-Associated Pneumonia Based the PERCH Integrated Analysis, Stratified by Age Group (**A**) and Study Site (**B**). The figures display the distribution of the individual case probability that rhinovirus was the cause of pneumonia based on the PERCH integrated etiology analysis [26]. Cases with an etiologic probability <5% for rhinovirus were excluded to scale the *y* axis and better visualize the cases with higher probability (>75%) of disease associated with rhinovirus. Cases testing positive for rhinovirus by nasopharyngeal/oropharyngeal RT-PCR are displayed in pink. Cases who tested negative by RT-PCR for rhinovirus are displayed in blue. Cases with missing nasopharyngeal/oropharyngeal PCR data are shown in gray.

Of the RV-positive cases, 13% (*N* = 105/912) had >75% probability that RV was the cause of their pneumonia based on the PERCH integrated etiology analysis [26]; 99% (N= 104/105) of these cases were in children over the age of 12 months from Bangladesh (Figure 2).

### 3.3. Case-Control Comparison of RV Infection in Children

**Figure 3:**
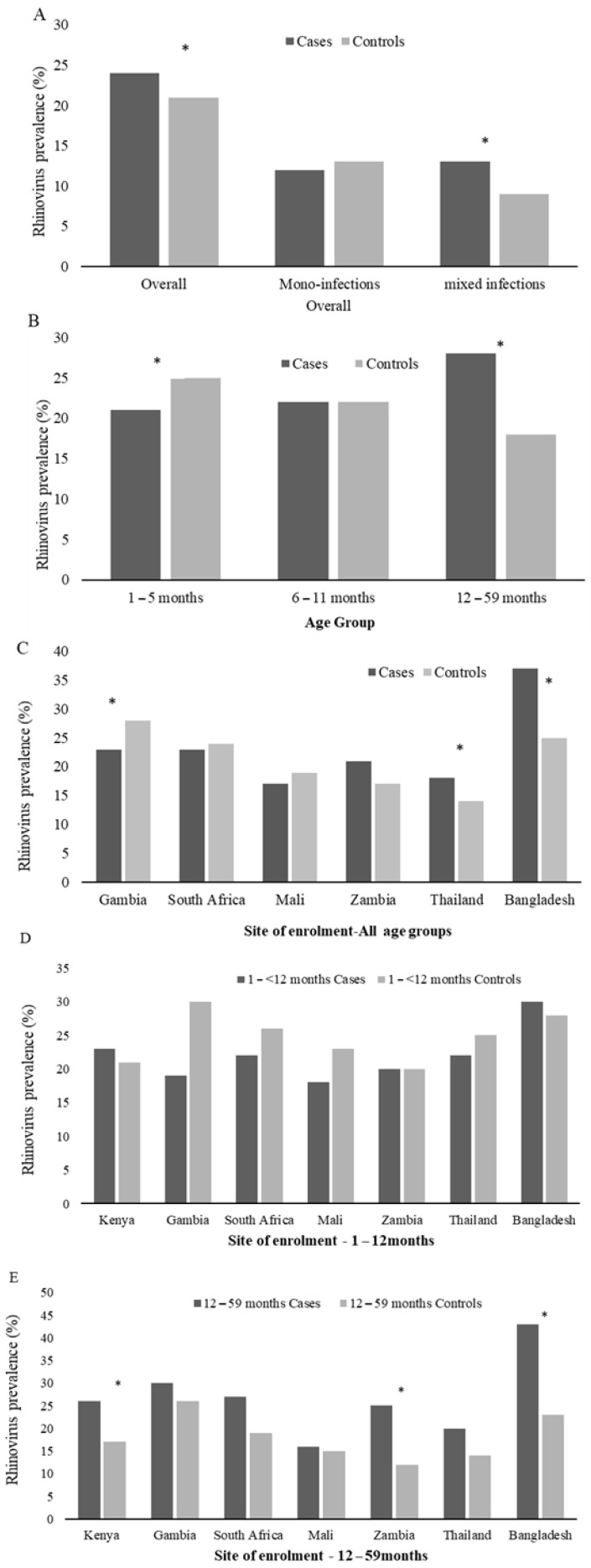
Prevalence of rhinovirus among Cases and Controls by PERCH overall (**A**), Age Group (**B**), and Site of enrolment (**C**–**E**). The * denotes groups where rhinovirus prevalence differs significantly (adjusted for site of enrolment and age where necessary) between cases and controls. Mono-infections refers to when rhinovirus was the only respiratory virus detected in the nasopharyngeal/oropharyngeal samples, and mixed infections refers to any viral co-infection with rhinovirus and RSV (**A**,**B**), HMPV, AdV, InFV (**A**–**C**), PIV type 1–4, or HCoV (OC43, NL63, 229E, and HKU1). By age group, RV detection was associated with control status in infants 1- to < 6-months of age, and RV detection was associated with case status in children >12–59 months of age. By site, RV detection was associated with case status in Bangladesh and Kenya but only in children >12 months of age.

In a multivariable analysis, adjusting for age category, site of enrolment and co-infecting bacteria and viruses, RV detection was more common among cases (24%) than controls (21%; aOR = 1.5, 95%CI:1.3–1.6). RV prevalence among cases varied by age group, with the highest prevalence among those 12–59 months of age (28%, Figure 3B). Further, the prevalence of RV among the 1- to 5-month age group cases was lower than the age group-matched controls (21% vs. 25%, *p* = 0.01), while higher among cases (28%) than controls (18%, aOR = 2.1, 95%CI:1.8–2.5; *p* = 0.03) in children over 12 months of age (Figure 3B and Supplementary Materials Table S5). Further, RV was more likely to be detected as a mixed viral infection among cases compared with controls (11% vs. 8%; *p* = 0.001) (Figure 3A and Supplementary Materials Table S5); however, by age group, this was only evident in the infants. The RV association with case status was only evident in Thailand, Bangladesh, and Kenya (Figure 3C and Supplementary Materials Table S5). However, when stratified to children >12 months, all sites showed a higher prevalence of RV detection in the cases vs. the controls—though mainly in Kenya, Zambia and Bangladesh (Figure 3D–E).

To identify risk factors for RV-positive pneumonia hospitalization, and because RV detection was associated with pneumonia in children >12 months of age (Figure 2B and Supplementary Materials Table S5), we compared RV-positive cases with RV-positive controls in children 12–59 months of age (Table 5). The only difference was that the RV-positive cases were more likely than controls to be underweight and to have higher NP/OP RV viral load (3.7 vs. 3.4 log10 copies/mL; *p* < 0.001), independent of whether RV was detected as a mono- (3.8 vs. 3.5 log10 copies/mL; *p* = 0.002) or co-infection (3.3 vs. 3.3 log10 copies/mL; *p* < 0.001) compared with the RV-positive controls. We were, however, unable to identify any RV viral load threshold that distinguished RV-positive cases from RV-infected controls either on the reverse cumulative plot or Youden index (Supplementary Materials Figure S2). Conversely, RV-positive cases were less likely to have a co-colonizing bacteria in the nasopharynx/oropharynx—in particular, *S. pneumoniae* and *M catarrhalis*. Similar trends were seen when RV-positive cases were compared with RV-positive controls, regardless of age (Supplementary Materials Table S6).

## 4. Discussion

In this large, multi-country, case-control pneumonia etiology study, it was found that children >12–59 months of age hospitalized with severe or very severe pneumonia were more likely to have RV detected on NP/OP swabs compared with community controls, even after adjustment for confounding variables, including the presence of other respiratory viruses. This association with case status was mainly observed in the Bangladesh sites. This association was, however, not observed among infants aged 1 to <12 months. Further, a high prevalence (20%) of RV detection was detected in non-ARI community controls concordant with other studies (10–24%) [9,13,15,16,18,29,30]. This is possibly due to widespread circulation of RV coupled with prolonged shedding of RV [31]. RV detection was significantly more common among controls with ARI (25%), once again mainly driven by the Asian sites. The association of RV with case status and ARI controls in Bangladesh children was the first real geographical difference in etiology of disease observed in the PERCH study and could potentially be linked to the increased incidence of wheezing disease and poorer air quality in Bangladesh [32]. Thus, although detection of RV in respiratory samples does not necessarily confirm causality with any concurrent illness/symptom, our results suggest a role of RV in the pathogenesis of some respiratory illness beyond the infancy period.

Notably, pneumonia cases where RV was the only respiratory virus detected in the nasopharyngeal/oropharyngeal sample in our study had higher viral loads, elevated CRP levels, and higher case fatality ratio compared with cases with RV and other viral co-infections after adjusting for site and age. This along with the negative association between RV and several other common respiratory viruses noted in our study suggests antagonism between the viruses and that perhaps when RV infections out compete other viruses, the result is a more severe infection. Regardless, the identification of RV alone among pneumonia cases is possibly of greater significance in attributing a role of RV to the pathogenesis or etiology of pneumonia among the cases. The association of RV mono-viral infections being associated with more severe disease was also reported in a previous South African study enrolling children less than 5 years of age with pneumonia [19]. Furthermore, cases had higher mean RV viral load than controls, and an association between increased viral load and more severe disease was observed among the RV-positive cases. However, we were unable to identify a specific RV nasopharyngeal density threshold to discriminate between cases and controls or among cases by severity. This association between higher RV viral load and more severe disease has also been described by others [33,34,35,36], where viral load correlated with illness severity.

When comparing RV-positive controls to RV-positive cases in children 12–59 months of age, the only risk factor associated with case status was malnutrition. This was, however, also a common case-control risk factor identified in the PERCH study [26]. Other study limitations included the cross-sectional study design with only a single specimen taken on enrollment into the study regardless of when symptoms started and that controls were not interviewed about disease episodes more than three days prior to enrollment. Thus, we were unable to determine the temporal association of detection of RV in relation to the onset of current or previous symptoms. Additionally, controls were not followed up to determine whether they became ill post sampling. Thus, we could not rule out the possibility they were in the incubation period of disease at the time of sampling. This was seen in a Finnish study [16] that enrolled children under the age of 15 years with acute wheezing (*N* = 161) as well as surgical controls (*N* = 79). They found that RV prevalence was 16% and 8% in the cases and controls, respectively. Although the controls were asymptomatic at the time of sampling, 5 of the 13 RV-positive controls developed respiratory symptoms in the following week [16]. Furthermore, our study used URT sampling as a proxy for sampling the site of infection. Direct sampling of the LRT, including lung aspirates and bronchoalveolar lavage, would provide more direct evidence on the causal pathogen of the pneumonia episode. However, these samples are invasive and difficult to perform in infants and children. The Fast Track multiplex PCR assay might have underestimated the RV prevalence, as the assay can fail to detect some RV strains [36,37]. We also cannot exclude cross-reactions between RV and enteroviruses. Genetic sequencing of positive specimens is required to better understand the contribution of RV species and enteroviruses to pneumonia etiology.

## 5. Conclusions

In conclusion, the large study size allowed us to analyze many different variables, interrogate the relevance of RV co-infections, and include controls to account for potential confounders for RV-positive disease severity and death. The study findings suggest that RV clinical outcomes are influenced by geographical location as well as by multiple host-specific factors, including age, nutritional status as well as RV viral loads and the presence of viral and bacterial co-infections. It also highlights the need to test for both viral and bacterial pathogens in children hospitalized with severe pneumonia. The risk factors for infection that we have identified point the way to interventions, but they are long-term development challenges that are not easy to correct. New treatment and prevention strategies are necessary to reduce what appears to be a substantial morbidity associated with RV disease.

## Figures and Tables

**Table 1:** Demographic and Clinical Characteristics and Respiratory Virus Co-infections among Community Controls with (*N* = 1056) and without (*N* = 3921) rhinovirus Infection, All PERCH Sites.

	Number		Percentage				
Characteristics	RV+	RV−	RV+	RV−	aOR ^a^	95%CI ^a^	*p*-Value ^a^
Demographic and health:							
12–59 months ^b^	409	1832	39	47	1.4	1.2–1.6	<0.001
Premature birth ^c^	130	376	12	10	1.3	1.1–1.7	0.01
Never breast fed	974	3583	93	92	1.2	0.9–1.7	0.16
Underweight ^d^	127	471	12	12	1.03	0.8–1.3	0.79
Male	538	1960	51	50	0.95	0.8–1.1	0.50
Day care attendance	163	739	16	19	0.95	0.8–1.1	0.50
Smoker in household	392	1518	37	39	0.91	0.8–1.1	0.15
Clinical:							
ARI ^e^	299	880	28	22	1.6	1.3–1.9	<0.001
Rhinorrhea	223	629	21	16	1.7	1.4–2.1	<0.001
Cough	120	300	11	8	1.6	1.2–2.0	<0.001
Fever ^f^	56	212	5	5	0.97	0.7–1.3	0.86
Tachypnea ^g^	103	454	10	12	0.8	0.6–1.0	0.05
Diarrhea	13	82	1	2	0.6	0.3–1.04	0.07
Respiratory viruses detected:				
AdV	134	458	13	12	1.2	0.98–1.5	0.07
HMPV	57	147	5	4	1.2	0.9–1.7	0.26
HBoV	138	521	13	13	1.02	0.8–1.3	0.80
PIV	68	246	6	6	0.9	0.7–1.2	0.63
HCoV	93	406	9	10	0.8	0.6–1.0	0.05
RSV	24	115	2	3	0.8	0.5–1.2	0.30
InFV A-C	9	104	1	3	0.3	0.1–0.5	<0.001
Any viral co-infection	400	183	38	47	0.7	0.6–0.8	<0.001
Bacterial infections in the NP/OP:							
*H. influenzae* type b	23	59	2	2	1.6	0.96–2.6	0.07
*M. catarrhalis*	836	2857	79	73	1.5	1.2–1.7	<0.001
*H. influenzae*	603	1958	57	50	1.4	1.2–1.6	<0.001
*S. pneumoniae*	860	2979	81	76	1.4	1.2–1.7	<0.001
*C. pneumoniae*	16	50	2	1	1.3	0.7–2.3	0.39
*M. pneumoniae*	18	52	2	1	1.3	0.8–2.3	0.31
*S. aureus*	135	542	13	14	0.9	0.7–1.1	0.16
*B. pertussis*	1	10	0	0	0.3	0.0–2.6	0.29
Any bacterial co-infection	1015	3687	96	94	1.7	1.2–2.3	0.003

Abbreviations—aOR: adjusted odds ratio; CI: confidence interval; ARI: acute respiratory infection; NP/OP: nasopharyngeal/oropharyngeal; RV: rhinovirus; RSV: respiratory syncytial virus; HMPV: human metapneumovirus; AdV: adenovirus; PIV: parainfluenza type 1–4; HBoV: human bocavirus; HCoV: human coronavirus (OC43, NL63, 229E and HKU1); and InFV: influenza virus (A, B and C). ^a^ *p*-values and aOR for being RV+ compared to RV− from regression models adjusted for age in months, site of enrollment, premature birth, breast feeding, smoker in the household, ARI status, and co-infecting bacteria and viruses where applicable. ^b^ The mean age and standard deviation (SD) for RV+ was 13.2 months (12.9), and RV− was 16.1 months (14.7), *p* < 0.001. ^c.^ Premature birth defined as gestational age <37 weeks. ^d^ Underweight defined as weight for age <−2SD of the median age-sex specific WHO reference. ^e^ Controls were considered to have ARI if they had (1) cough or runny nose or (2) one of the following signs: ear discharge, wheeze, or difficulty breathing, in the presence of sore throat or fever (temperature ≥38.0 °C or reported fever in the past 48 h). ^f^ Fever defined as temperature ≥38 °C or reported fever in the past 48 h. ^g^ Tachypnea defined as respiratory rate ≥60 breaths/minute if aged <2 months, respiratory rate ≥50 breaths/minute if aged 2–12 months, respiration rate ≥40 breaths/minute if aged >12 months.

**Table 2:** Demographic and Clinical Characteristics of Community Controls with rhinovirus as the Only Detected Virus (Mono-RV; *N* = 656) and Those with rhinovirus Plus at Least One Other Respiratory Virus Detected (Mixed-RV; *N* = 400), All PERCH Sites.

	Number		Percentage				
	Mono-Rv Infections	Mixed-Rv Infections ^a^	Mono-Rv Infections	Mixed-Rv Infections ^a^	aOR ^b^	95%CI ^b^	*p*-Value ^b^
Demographic and health:							
12–59 months of age ^c^	228	181	35	45	1.6	1.3–1.9	<0.001
Smoker in household	243	149	37	38	1.2	0.9–1.5	0.31
Male	335	206	51	51	1.01	0.8–1.3	0.92
Never breast fed	52	25	8	6	1.01	0.6–1.8	0.97
Underweight ^d^	75	52	11	13	0.98	0.7–1.5	0.91
Premature birth^e^	92	38	14	10	0.95	0.9–1.1	0.44
Day care attendance	86	77	13	19	0.9	0.7–1.2	0.35
Clinical Features:							
ARI ^f^	176	124	27	31	0.96	0.7–1.3	0.77
Rhinorrhea	132	733	20	17	1.08	0.9–1.2	0.22
Fever ^g^	32	24	5	6	1.06	0.6–1.9	0.85
Cough	68	52	10	13	0.93	0.8–1.1	0.38
Tachypnea ^h^	62	41	10	10	0.9	0.6–1.4	0.68
Diarrhea	10	3	2	1	0.8	0.5–1.1	0.20
Bacterial infections in the NP/OP:							
*H. influenzae* type b	13	8	2	2	1.4	0.7–2.5	0.32
*S. pneumoniae*	536	304	82	76	1.4	1.2–1.8	0.001
*C. pneumoniae*	10	5	2	1	1.3	0.6–2.5	0.50
*M. catarrhalis*	502	296	77	74	1.2	0.95–1.4	0.12
*H. influenzae*	336	206	52	51	1.0	0.9–1.3	0.65
*S. aureus*	92	54	14	14	0.98	0.8–1.3	0.89
*B. pertussis*	1	1	0	0	0.5	0.1–4.3	0.57
*M. pneumoniae*	5	7	1	2	0.5	0.2–1.3	0.16
Any bacterial co-infection	628	370	96	92	2.2	1.5–3.2	<0.001

Abbreviations—aOR: adjusted odds ratio; CI: confidence interval; RV: rhinovirus; ARI—acute respiratory infection. ^a^ Any viral respiratory coinfection with rhinovirus and respiratory syncytial virus (A or B), human metapneumovirus, adenovirus, influenza virus (A, B or C), parainfluenza virus type 1–4, human coronavirus (OC43, NL63, 229E or HKU1). ^b^ *p*-values and aOR for having a mono-RV infection compared with a mixed-RV infection from regression models adjusted for age in months, site of enrollment, and co-infecting bacteria where applicable. ^c^ The mean age and standard deviation (SD) for mono-RV infections was 12.4 months (SD: 12.8 months), and mixed-RV infection was 14.5 months (SD:13.0 months; *p* = 0.04). ^d^ Underweight defined as weight for age <−2SD of the median age-sex specific WHO reference. ^e^ Premature birth defined as gestational age <37 weeks. ^f^ Controls were considered to have acute respiratory tract illness (ARI) if they had (1) cough or runny nose or (2) one of the following signs: ear discharge, wheeze, or difficulty breathing, in the presence of sore throat or fever (temperature ≥38.0 °C or reported fever in the past 48 h). ^g^ Fever defined as temperature ≥38 °C. ^h^ Tachypnea defined as respiratory rate ≥60 breaths/minute if aged <2 months, respiratory rate ≥50 breaths/minute if aged 2–12 months, respiration rate ≥40 breaths/minute if aged >12 months.

**Table 3:** Demographic and Clinical Characteristics and Co-Infections among Pneumonia Cases with (*N* = 912) and without (*N* = 2958) rhinovirus Infection, All PERCH Sites.

	Number		Percentage				
	RV+	RV−	RV+	RV−	aOR ^a^	95% CI ^a^	*p*-Value ^a^
Demographic and health:							
12–59 months of age ^b^	393	1025	43	35	0.7	0.6–0.9	<0.001
Smoker in household	340	969	37	33	1.1	0.96–1.4	0.12
Male	539	1701	59	58	1.1	0.9–1.2	0.49
Day Care attendance	126	517	14	17	0.96	0.7–1.3	0.77
Underweight ^c^	277	916	30	31	0.9	0.8–1.1	0.41
Premature birth ^d^	87	328	10	11	0.9	0.7–1.1	0.31
Never breast fed	83	330	9	11	0.9	0.7–1.1	0.29
Clinical features:							
Wheezing	421	897	46	31	1.8	1.4–2.2	<0.001
Tachypnea ^e^	785	2379	86	81	1.5	1.1–1.9	0.01
Very severe pneumonia	291	955	32	32	1.1	0.96–1.4	0.13
Deaths ^f^	47	185	5	6	1.0	0.7–1.4	0.98
Diarrhea	118	451	13	15	1.0	0.8–1.3	0.99
Tachycardia ^g^	439	1512	48	51	0.98	0.8–1.2	0.83
Hypoxic ^h^	297	1086	33	37	0.98	0.8–1.2	0.85
Chest X-ray abnormal ^i^	365	1354	0	46	0.8	0.7–1.0	0.05
Hospital stay > 3 days	453	1729	50	58	0.8	0.7–0.97	0.02
Convulsions	43	201	5	7	0.7	0.5–1.04	0.08
Any symptom	909	2940	99	99	1.9	0.5–6.3	0.99
Bacterial infection markers:							
Leukocytosis ^j^	443	1133	51	41	1.3	1.1–1.5	0.01
Blood culture positive ^k^	29	107	3	4	0.99	0.6–1.5	0.96
Fever ^l^	707	2441	78	83	0.96	0.8–1.2	0.78
CRP > 40 mg/mL ^m^	176	715	19	24	0.9	0.7–1.1	0.17
Alveolar consolidation	156	657	18	24	0.8	0.7–1.1	0.13
MCPP ^n^	8	35	1	1	0.8	0.4–1.8	0.66
Any bacterial marker	827	2701	91	91	0.9	0.7–1.2	0.56
Respiratory viral infections in the NP/OP:							
AdV	108	282	12	10	1.3	0.98–1.6	0.07
HCoV	56	232	6	8	0.8	0.6–1.1	0.14
HMPV	49	293	5	10	0.5	0.4–0.7	<0.001
PIV	76	435	8	15	0.4	0.3–0.5	<0.001
HBoV	138	364	15	12	0.3	0.2–0.5	<0.001
RSV	121	832	13	28	0.3	0.2−0.4	<0.001
InFV A-C	5	173	1	6	0.1	0.02–0.2	<0.001
Any viral co-infection	431	2192	47	74	0.3	0.25–0.34	<0.001
Bacterial infections in the NP/OP:							
*H. influenzae* type b	23	57	3	2	1.4	0.8–2.3	0.19
*B. pertussis*	8	23	1	1	1.3	0.6–3.0	0.48
*C. pneumoniae*	9	26	1	1	1.2	0.5–2.5	0.73
*M. catarrhalis*	616	1944	68	66	1.1	0.96–1.3	0.15
*H. influenzae*	510	1567	56	53	1.1	0.9–1.3	0.23
*S. pneumoniae*	661	2117	72	72	1.04	0.9–1.2	0.66
*S. aureus*	129	494	14	17	0.86	0.7–1.1	0.18
*M. pneumoniae*	12	45	1	2	0.8	0.4–1.6	0.60
Any bacterial co-infection	845	2714	93	92	0.5	0.8–1.5	0.53

Abbreviations—aOR: adjusted odds ratio; CI: confidence interval; NP/OP: nasopharyngeal/oropharyngeal; RV: rhinovirus; CRP: C-reactive protein; MCPP: microbiologically confirmed pneumococcal pneumonia; RSV: respiratory syncytial virus; HMPV: human metapneumovirus; AdV: adenovirus; PIV: parainfluenza type 1–4; HBoV: human bocavirus; HCoV: human coronavirus (OC43, NL63, 229E, and HKU1); and InFV: influenza virus (A, B and C). *S. aureus: Staphylococcus aureus; S. pneu: Streptococcus pneumoniae*; *H. influenzae: Haemophilus influenzae, H. influenzae* type; *M. catarrhalis: Moraxella catarrahalis; B. pertussis: Bordetella pertussis; M. pneumoniae: Mycoplasma pneumoniae; C. pneumoniae: Chlamydia pneumoniae.* ^a^ *p*-values and aOR for being RV+ compared with RV− from regression models adjusted for age in month, site of enrollment, smoker in the household, severity of pneumonia diagnosis, and co-infecting bacteria and viruses where applicable. ^b^ The mean age and standard deviation (SD) for RV+ cases was 13.1 months (SD:12.3 months) and RV− cases (11.2 months (SD: 11.3; *p* = 0.50). ^c^ Underweight defined as weight for age <−2SD of the median age-sex specific WHO reference. ^d^ Premature birth defined as gestational age <37 weeks. ^e^ Tachypnea defined as respiratory rate ≥60 breaths/minute if aged <2 months, respiratory rate ≥50 breaths/minute if aged 2–12 months, respiration rate ≥40 breaths/minute if aged >12 month. ^f^ Died while in hospital. ^g^ Tachycardia defined as heart rate >160 beats per minute (bpm) if aged <11 months, heart rate >150 bpm if aged 12–35 months, heart rate >140 bpm if aged 36–59 months. ^h^ A child was considered to be hypoxic if (1) a room air pulse-oximetry reading indicated oxygen saturation <90% at the two sites at elevation (Zambia and South Africa) or <92% at all other sites or (2) a room air oxygen saturation was not available, and the child was placed on supplemental oxygen. ^i^ Abnormal chest X-ray defined as radiographically confirmed end point pneumonia consolidation or any infiltrates. ^j^ Leukocytosis defined as white blood cell count >15,000 cells/µL if age <12 months or >13,000 cells/µL if age >12 months. ^k^ Blood culture positive for any non-contaminate bacteria. ^l^ Fever defined as temperature ≥38 °C. ^m^ CRP defined as levels ≥40 mg/mL are considered to potentially indicate bacterial infection. ^n^ MCPP defined as *S.* *pneumoniae* was cultured from a normally sterile body fluid (blood, pleural fluid, or lung aspirate), or pleural fluid or lung aspirate was PCR *LytA* positive.

**Table 4:** Demographics and Clinical Characteristics of Severe and Very-Severe Pneumonia Hospitalized Cases With rhinovirus As the Only Detected Virus (Mono-RV; *N* = 481) and Those With rhinovirus Plus at Least One Other Respiratory Virus Detected (Mixed-RV; *N* = 431).

	Number		Percentage				
	Mono-RV Infections	Mixed-RV Infections ^a^	Mono-RV Infections	Mixed-RV Infections ^a^	aOR	95%CI ^b^	*p*-Value
Demographic and health:							
12–59 months of age ^c^	231	162	48	38	0.6	0.5–0.8	<0.001
Never breast fed	49	34	10	8	1.3	0.8–2.2	0.28
Premature birth ^d^	57	30	12	7	1.3	0.8–1.95	0.28
Underweight ^e^	156	121	32	28	1.1	0.9–1.5	0.38
Male	285	254	59	59	1.1	0.8–1.4	0.59
Day Care attendance	60	66	12	15	1.1	0.6–1.8	0.79
Smoker in household	163	177	34	41	0.95	0.7–1.3	0.76
Clinical features:							
Deaths ^f^	33	14	7	3	2.6	1.2–5.5	0.01
Convulsions	30	13	6	3	1.98	0.98–4.0	0.06
Diarrhea	67	51	14	12	1.2	0.8–1.9	0.31
Tachycardia ^g^	199	240	46	50	1.1	0.8–1.4	0.57
Very severe pneumonia	163	128	34	30	1.1	0.8–1.5	0.71
Wheezing	214	207	45	48	0.99	0.7–1.4	0.95
Chest X-ray abnormal ^h^	188	177	39	41	0.9	0.7–1.2	0.46
Hospital stay >3 days	233	220	48	51	0.9	0.7–1.2	0.45
Hypoxic ^i^	156	141	33	33	0.8	0.6–1.1	0.22
Tachypnea ^j^	380	405	89	84	0.8	0.5–1.1	0.18
Any symptom	480	429	100	100	2.2	0.2–24.8	0.51
Bacterial co-infection markers:							
MCPP ^k^	6	2	1	0	4.3	0.8–22.4	0.08
Blood culture positive ^l^	18	11	4	3	1.9	0.9–4.3	0.12
CRP ≥40 mg/mL ^m^	108	68	22	16	1.6	1.0–2.4	0.04
Leukocytosis ^n^	256	187	55	47	1.3	0.9–1.7	0.13
Fever ^o^	369	338	77	78	1.02	0.7–1.4	0.91
Alveolar consolidation	78	78	17	19	0.9	0.6–1.3	0.53
Any marker of bacterial infection	435	392	90	91	0.8	0.6–1.5	0.79
Bacterial infections in the NP/OP:							
*B. pertussis*	6	2	1	0	3.0	0.6–15.2	0.18
*S. aureus*	76	53	16	12	1.4	0.98–2.1	0.06
*C. pneumoniae*	5	4	1	1	0.99	0.3–3.8	0.99
*M. catarrhalis*	313	303	65	70	0.7	0.5–0.98	0.04
*H. influenzae*	244	266	51	61	0.6	0.5–0.8	0.001
*S. pneumoniae*	338	323	70	75	0.6	0.6–1.01	0.07
*H. influenzae* type b	9	14	2	3	0.5	0.2–1.3	0.16
*M. pneumoniae*	4	8	1	2	0.4	0.1–1.5	0.18
Any bacterial co-infection	440	405	91	94	0.7	0.4–1.2	0.13

Abbreviations—aOR: adjusted odds ratio; CI: confidence interval; SD: standard deviation; RV: rhinovirus; CRP: C-reactive protein; MCPP: microbiologically confirmed pneumococcal pneumonia. ^a^ Any viral respiratory coinfection with rhinovirus and respiratory syncytial virus (A or B), human metapneumovirus, adenovirus, influenza virus (A, B or C), parainfluenza virus type 1–4, human coronavirus (OC43, NL63, 229E or HKU1). ^b^ *p*-values and aOR for having a mono-RV infection compared with a mixed-RV infection from regression models adjusted for age in month, site of enrollment, and co-infecting bacteria where applicable. ^c^ The mean age and standard deviation (SD) for mono-RV infections was 14.2 months (SD: 12.6 months), and mixed-RV infection was 11.9 months (SD:11.3 months; *p* = 0.005). ^d^ Premature birth defined as gestational age <37 weeks. ^e^ Underweight defined as weight for age <−2SD of the median age-sex specific WHO reference. ^f^ Died while in hospital. ^g^ Tachycardia defined as heart rate >160 beats per minute (bpm) if aged <11 months, heart rate >150 bpm if aged 12–35 months, heart rate >140 bpm if aged 36–59 months. ^h^ Abnormal chest X-ray defined as radiographically confirmed end point pneumonia consolidation or any infiltrates. ^i^ A child was considered to be hypoxic if (1) a room air pulse-oximetry reading indicated oxygen saturation <90% at the two sites at elevation (Zambia and South Africa) or <92% at all other sites or (2) a room air oxygen saturation was not available, and the child was placed on supplemental oxygen. ^j^ Tachypnea defined as respiratory rate ≥60 breaths/minute if aged <2 months, respiratory rate ≥50 breaths/minute if aged 2–12 months, respiration rate ≥40 breaths/minute if aged >12 month. ^k^ MCPP defined as *S.* *pneumoniae* was cultured from a normally sterile body fluid—blood, pleural fluid, or lung aspirate—or pleural fluid or lung aspirate was PCR *LytA* positive. ^l^ Blood culture positive for any non-contaminate bacteria. ^m^ CRP defined as levels ≥40 mg/mL are considered to potentially indicate bacterial infection. ^n^ Leukocytosis defined as white blood cell count >15,000 cells/µL if age <12 months or >13,000 cells/µL if age >12 months. ^o^ Fever defined as temperature ≥38 °C.

**Table 5:** Demographic, Clinical, and Laboratory Findings of 12- to 59-month-old rhinovirus-Positive Pneumonia Cases (*N* = 393) and Controls (*N* = 409), All PERCH Sites.

	Number		Percentage				
	RV+ cases	RV+ controls	RV+ cases	RV+ controls	aOR ^a^	95%CI ^a^	*p*-Value ^a^
Demographic and health:							
Underweight ^b^	140	66	36	16	2.8	2.0–4.0	<0.001
Male	175	198	45	48	0.9	0.7–1.2	0.338
Never breast fed	32	30	8	7	1.5	0.8–2.7	0.213
Smoker in household	161	162	41	40	1.1	0.8–1.4	0.673
Day Care attendance	54	85	14	21	0.7	0.4–1.2	0.216
Premature birth ^c^	32	48	8	12	0.8	0.5–1.3	0.428
RV epidemiology:							
RV Co-infections ^d^	162	181	41	44	0.9	0.7–1.2	0.526
RV Mono-infection ^e^	231	228	59	56	1.1	0.8–1.5	0.526
Respiratory viral co-infections in the NP/OP:							
RSV	17	6	4	1	3.2	1.2–8.2	0.018
PIV	24	33	6	8	0.7	0.4–1.3	0.245
HBoV	70	68	18	17	1.1	0.8–1.7	0.507
HMPV	147	26	4	6	0.7	0.3–1.3	0.243
AdV	59	81	15	20	0.7	0.5–1.1	0.105
InFV A-C	2	1	1	0	2.1	0.2–23	0.549
HCoV	20	34	5	8	0.6	0.3–1.0	0.069
Bacterial infections in the NP/OP:							
*B. pertussis*	2	0	1	0	-	-	0.149
*H. influenzae* type b	10	12	3	3	0.8	0.4–2.0	0.701
*S. aureus*	41	34	10	8	1.4	0.8–2.2	0.193
*H. influenzae*	222	246	56	60	0.8	0.6–1.1	0.150
*M. pneumoniae*	7	12	2	3	0.6	0.2–1.5	0.288
*C. pneumoniae*	7	7	2	2	1.1	0.4–3.2	0.874
*S. pneumoniae*	285	339	73	83	0.5	0.4–0.7	<0.001
*M. catarrhalis*	261	330	66	81	0.4	0.3–0.6	<0.001
Any bacterial co-infection	366	397	93	97	0.4	0.2–0.8	0.010

Abbreviations—aOR: adjusted odds ratio; CI: confidence interval; SD: standard deviation; NP/OP nasopharyngeal/oropharyngeal; RV: rhinovirus; RSV: respiratory syncytial virus; HMPV: human metapneumovirus; AdV: adenovirus; PIV: parainfluenza type 1–4; HBoV: human bocavirus; HCoV: human coronavirus (OC43, NL63, 229E, and HKU1); and InFV: influenza virus (A, B and C). ^a^ *p*-values and aOR for being a RV+ case compared with a RV+ control from regression models adjusted for age in month, site of enrollment, prematurity, sex, breastfeeding practices, co-infecting viruses, and bacteria where applicable. ^b^ Underweight defined as weight for age <−2SD of the median age-sex specific WHO reference. ^c^ Premature birth defined as gestational age <37 weeks. ^d^ Any viral respiratory coinfection with RSV (A and B), HMPV, AdV, InFV (A, B and C), PIV type 1–4, HCoV (OC43, NL63, 229E, and HKU1). ^e^ RV was the only respiratory virus detected in the nasopharyngeal/oropharyngeal sample.

## Data Availability

All data are available at https://clinepidb.org/ce/app (accessed 12 May 2021).

## Supplementary Materials

The following are available online at https://www.mdpi.com/article/10.3390/v13071249/s1, Figure S1: Reverse Cumulative Plot of RV Viral Load in the Nasopharyngeal/Oropharyngeal Sample among Very Severe and Severe Pneumonia Cases. The Viral Loads of Very Severe Cases Are Shown in Red and the Severe Cases in Purple, Figure S2: Reverse Cumulative Plot of RV Viral Load in the Nasopharyngeal/Oropharyngeal Sample among Cases and Controls. The Viral Loads of Cases Are Shown in Red and the Controls in Blue. Table S1: Demographic and Clinical Characteristics of Controls by rhinovirus Infection Status for Each African Site, Table S2: The Demographic and Clinical Characteristics of Controls by rhinovirus Infection Status for Each Southeast Asian Site Individually, Table S3: The Demographic and Clinical Characteristics of Cases by rhinovirus Infection Status for Each African Site Individually, Table S4: The Demographic and Clinical Characteristics of Cases by rhinovirus Infection Status for Each Southeast Asia Site Individually, Table S5: rhinovirus-Positive Pneumonia Cases (*N* = 912) and Controls (*N* = 1056) overall, by site of enrolment, and by age group, Table S6: Demographic, Clinical and Laboratory Findings of rhinovirus-Positive Pneumonia Cases (*N* = 912) and Controls (*N* = 1056), All PERCH Sites.
