# Supplementary material for: Epidemiology of the Rhinovirus (RV) in African and Southeast Asian Children: A Case-Control Pneumonia Etiology Study
Source: Viruses. 2021 Jun 27;13(7):1249. doi: 10.3390/v13071249 (PMC8310211; doi:10.3390/v13071249)
Supplement: Supplementary file 1 [file viruses-13-01249-s001.zip › viruses-1238670-supplementary.pdf]

Table S1: Demographic and Clinical Characteristics of Controls by Rhinovirus Infection Status for Each African Site.

Characteristics	Kenya (n=855)			Gambia (n=624)			Mali (n=724)			Zambia (n=533)			South Africa (n=823)		
	RV+ (n=162) N (%)	RV- (n=693) N (%)	*P*-value[a]	RV+ (n=177) N (%)	RV- (n=447) N (%)	*P*-value[a]	RV+ (n=143) N (%)	RV- (n=581) N (%)	*P*-value[a]	RV+ (n=93) N (%)	RV- (n=440) N (%)	*P*-value[a]	RV+ (n=194) N (%)	RV- (n=629) N (%)	*P*-value[a]
<u>Demographic and health:</u>															
Mean Age, months (SD)	14.8 (14.3)	17.3 (14.7)	0.05	14.1 (13.5)	16.9 (14.7)	0.03	10.0 (9.6)	14.7 (13.5)	*P*<0.001	6.9 (6.2)	10.5 (11.3)	0.01	11.3 (11.3)	12.6 (12.6)	0.21
Female	85 (52)	319 (46)	0.16	75 (42)	220 (49)	0.12	62 (43)	304 (52)	0.05	36 (39)	226 (51)	0.03	106 (55)	317 (50)	0.26
Never breast fed	1 (1)	6 (1)	0.86	1 (1)	1 (0)	0.49	0	3 (1)	0.84	2 (2)	13 (3)	0.62	66 (34)	222 (35)	0.79
Under weight[b]	26 (16)	99 (14)	0.29	23 (13)	79 (18)	0.24	18 (13)	61 (11)	0.26	9 (10)	56 (13)	0.74	10 (5)	27 (4)	0.60
Day Care attendance	5 (3)	42 (6)	0.14	0	11 (2)	0.05	107 (75)	445 (77)	0.66	0	15 (3)	0.17	28 (14)	93 (15)	0.98
Smoker in household	43 (27)	214 (31)	0.27	99 (58)	248 (56)	0.70	26 (18)	122 (21)	0.66	28 (30)	122 (27)	0.64	48 (30)	198 (32)	0.46
Premature birth[c]	32 (20)	75 (11)	0.01	11 (6)	22 (5)	0.65	5 (4)	7 (1)	0.05	3 (3)	28 (6)	0.23	66 (34)	184 (29)	0.17
Birth weight, mean (SD)	3.1 (0.6)	3.1 (0.6)	0.50	3.1 (0.6)	2.9 (0.5)	0.17	3.3 (0.6)	3.3 (0.7)	0.86	2.9 (0.4)	2.9 (0.5)	0.96	3.0 (0.6)	3.0 (0.5)	0.19
<u>Clinical Features:</u>															
ARI [d]	50 (31)	160 (23)	0.05	52 (29)	104 (23)	0.12	61 (43)	237 (41)	0.33	10 (10)	43 (10)	0.58	11 (6)	34 (5)	0.97
Tachypnea[e]	31 (19)	103 (15)	0.25	23 (13)	58 (13)	0.94	20 (14)	98 (17)	0.44	6 (6)	58 (13)	0.15	11 (6)	66 (11)	0.06
Cough	16 (10)	26 (4)	0.01	21 (12)	42 (9)	0.35	12 (8)	48 (8)	0.95	7 (8)	29 (7)	0.47	5 (3)	10 (2)	0.40
Fever[f]	8 (5)	25 (4)	0.37	25 (14)	59 (13)	0.73	2 (1)	16 (3)	0.36	0	4 (1)	0.66	0	5 (1)	0.41
Diarrhoea	2 (1)	14 (2)	0.51	6 (3)	18 (4)	0.75	15 (3)	1 (1)	0.21	1 (1)	7 (2)	0.85	0	2 (0)	0.57
Rhinorrhoea	39 (24)	119 (17)	0.04	25 (14)	45 (10)	0.16	52 (36)	196 (34)	0.22	3 (3)	11 (3)	0.75	2 (1)	12 (2)	0.36

Abbreviations - SD: standard deviation; RV: rhinovirus; ARI: acute respiratory infection.

a. *P*-values comparing RV+ and RV- controls from regression models adjusted for age in months and site of enrollment and for confounding covariates where applicable. *P*-values could not be calculated for variables where both values are 0, thus cells left blank.
b. Underweight defined as weight for age <-2SD of the median age-sex specific WHO reference.
c. Premature birth defined as gestational age <37 weeks.
d. Controls were considered to have ARI if they had 1) cough or runny nose, or 2) one of the following signs, ear discharge, wheeze, or difficulty breathing, in the presence of sore throat or fever (temperature ≥38.0°C or reported fever in the past 48 hours).
e. Tachypnea defined as respiratory rate >60 breaths/minute if aged <2 months, respiratory rate >50 breaths/minute if aged 2-12 months, respiration rate >40 breaths/minute if aged >12 month.
f. Fever defined as temperature >38°C.

Table S2: The Demographical and Clinical Characteristics of Controls by Rhinovirus Infection Status for Each Southeast Asian Site Individually.

**Characteristics**	**Thailand (n=650)**			**Bangladesh (n=768)**		
	**RV+ (n=92) N (%)**	**RV- (n=558) N (%)**	***P*-value[a]**	**RV+ (n=195) N (%)**	**RV- (n=573 N (%))**	***P*-value[a]**
Demographic and health:						
Mean Age, months (SD)	20.7 (15.5)	21.1 (15.9)	0.82	14.9 (13.6)	18.5 (16.3)	0.01
Female	49 (53)	271 (49)	0.39	102 (53)	300 (52)	0.92
Never breast fed	5 (5)	65 (12)	0.08	2 (1)	2 (0)	0.23
Under weight[b]	4 (4)	33 (6)	0.52	37 (19)	116 (20)	1.00
Day Care attendance	22 (24)	132 (24)	0.96	1 (1)	1 (0)	0.61
Smoker in household	57 (62)	327 (59)	0.54	81 (42)	287 (50)	0.05
Premature birth[c]	10 (11)	40 (7)	0.02	3 (2)	20 (3)	0.36
Birth weight, mean (SD)	3.1 (0.4)	3.0 (0.5)	0.46	2.9 (0.6)	2.9 (0.5)	0.96
Clinical Features:						
ARI[d]	60 (65)	190 (34)	<0.001	56 (29)	113 (20)	0.02
Tachypnea[e]	2 (2)	46 (8)	0.07	10 (5)	28 (5)	0.89
Cough	27 (29)	81 (15)	0.01	32 (16)	63 (11)	0.06
Fever[f]	13 (14)	73 (13)	0.83	8 (4)	30 (5)	0.50
Diarrhoea	2 (2)	16 (3)	0.78	1 (1)	10 (2)	0.20
Rhinorrhoea	59 (64)	175 (31)	P<0.001	43 (22)	71 (12)	0.02

Abbreviations - SD: standard deviation; RV: rhinovirus; ARI: acute respiratory infection.

a. *P*-values comparing RV+ and RV- controls from regression models adjusted for age in months and site of enrollment and for confounding covariates where applicable.
b. Underweight defined as weight for age <-2SD of the median age-sex specific WHO reference.
c. Premature birth defined as gestational age <37 weeks.
d. Controls were considered to have ARI if they had 1) cough or runny nose, or 2) one of the following signs, ear discharge, wheeze, or difficulty breathing, in the presence of sore throat or fever (temperature ≥38.0°C or reported fever in the past 48 hours).
e. Tachypnea defined as respiratory rate >60 breaths/minute if aged <2 months, respiratory rate >50 breaths/minute if aged 2-12 months, respiration rate >40 breaths/minute if aged >12 month.
f. Fever defined as temperature >38°C.

Table S3: The Demographical and Clinical Characteristics of Cases by Rhinovirus Infection Status for Each African Site Individually.

Characteristics	**Kenya (n=628)**			**Gambia (n=609)**			**Mali (n=650)**			**Zambia (n=449)**			**South Africa (n=794)**		
	**RV+ (n=152) N (%)**	**RV- (n=476) N (%)**	***P*-value[a]**	**RV+ (n=139) N (%)**	**RV- (n=470) N (%)**	***P*-value[a]**	**RV+ (n=112) N (%)**	**RV- (n=538) N (%)**	***P*-value[a]**	**RV+ (n=95) N (%)**	**RV- (n=354) N (%)**	***P*-value[a]**	**RV+ (n=182) N (%)**	**RV- (n=794) N (%)**	***P*-value[a]**
<u>Demographic and health:</u>															
Mean Age, months (SD)	14.5 (13.2)	14.2 (13.1)	0.80	14.4 (12.5)	11.3 (11.2)	0.01	9.3 (10.1)	10.0 (10.2)	0.53	9.9 (10.9)	7.6 (8.9)	0.04	9.9 (9.7)	8.5 (9.5)	0.08
Male	60 (39)	201 (42)	0.52	55 (40)	176 (37)	0.97	50 (45)	237 (44)	0.90	47 (49)	158 (45)	0.44	80 (44)	291 (48)	0.41
Never breast fed	3 (2)	6 (1)	0.48	3 (2)	4 (1)	0.29	7 (6)	25 (5)	0.38	4 (4)	22 (6)	0.51	52 (29)	211 (34)	0.15
Under weight[c]	60 (39)	190 (43)	0.81	41 (30)	119 (25)	0.90	37 (33)	195 (36)	0.70	34 (36)	87 (25)	0.05	37 (20)	174 (28)	0.03
Day care attendance	8 (5)	29 (6)	0.57	2 (1)	11 (2)	0.51	83 (74)	350 (65)	0.17	1 (1)	5 (1)	0.84	24 (13)	92 (15)	0.38
Smoker in household	1 (1)	10 (2)	0.24	79 (57)	251 (54)	0.57	33 (30)	153 (28)	0.84	27 (28)	61 (17)	0.05	73 (40)	217 (35)	0.50
Premature birth[d]	28 (18)	87 (18)	0.62	5 (4)	19 (4)	0.94	3 (3)	25 (5)	0.56	2 (2)	21 (6)	0.28	33 (18)	119 (19)	0.79
<u>Clinical features:</u>															
Very severe pneumonia	80 (53)	242 (51)	0.76	25 (18)	63 (13)	0.32	65 (58)	273 (51)	0.15	32 (34)	108 (31)	0.58	65 (36)	189 (31)	0.21
Chest X-ray abnormal[e]	65 (43)	217 (46)	0.51	58 (42)	215 (46)	0.40	34 (30)	205 (38)	0.15	32 (34)	148 (42)	0.15	85 (47)	344 (56)	0.02
Hypoxic[f]	48 (32)	136 (29)	0.32	7 (5)	36 (8)	0.28	57 (51)	249 (46)	0.41	29 (31)	133 (38)	0.26	135 (75)	457 (75)	0.90
Tachycardia[g]	77 (51)	270 (57)	0.20	65 (47)	264 (56)	0.04	69 (62)	300 (56)	0.31	64 (67)	220 (63)	0.39	87 (48)	299 (49)	0.84
Tachypnea[h]	115(76)	322 (68)	0.06	121 (87)	409 (87)	0.81	101 (90)	450 (84)	0.06	84 (88)	302 (86)	0.74	150 (83)	459 (77)	0.10
Wheezing	31 (21)	52 (11)	0.01	48 (35)	142 (30)	0.14	27 (24)	87 (16)	0.07	21 (22)	35 (10)	0.01	79 (44)	188 (32)	0.01
Fever[i]	119(78)	416 (87)	0.01	135 (97)	466 (99)	0.05	95 (85)	472 (88)	0.53	73 (77)	300 (85)	0.04	116 (64)	382 (62)	0.94
Convulsions	14 (9)	61 (13)	0.15	8 (6)	18 (4)	0.55	11 (10)	73 (14)	0.37	4 (4)	17 (5)	0.62	5 (3)	5 (1)	0.05
Diarrhoea	25 (16)	72 (15)	0.77	13 (9)	41 (9)	0.64	25 (22)	140 (26)	0.47	22 (23)	69 (19)	0.87	30 (16)	89 (15)	0.40
Leucocytosis[j]	77 (52)	203 (43)	0.11	50 (48)	141 (41)	0.43	28 (25)	156 (29)	0.57	43 (46)	141 (41)	0.56	93 (51)	260 (43)	0.07
CRP >40mg/l[k]	29 (19)	125 (26)	0.05	25 (18)	104 (22)	0.18	30 (27)	159 (30)	0.76	31 (33)	107 (30)	0.80	38 (21)	161 (26)	0.07
Blood culture positive[l]	3 (2)	9 (2)	0.80	4 (3)	23 (5)	0.21	11 (10)	28 (5)	0.06	6 (6)	22 (6)	1.0	3 (2)	16 (3)	0.52
MCPP[m]	1 (1)	4 (1)	0.81	3 (2)	12 (3)	0.67	4 (4)	14 (3)	0.51	0	2 (1)	0.84	0	3 (0)	0.62
Hospital stay >3 days	25 (16)	106 (22)	0.16	10 (7)	45 (20)	0.45	47 (42)	266 (49)	0.13	38 (40)	131 (37)	0.47	77 (42)	358 (59)	<0.001
Case fatality ratio	9 (6)	27 (6)	0.79	7 (5)	14 (3)	0.33	25 (23)	87 (17)	0.13	14 (26)	55 (28)	0.78	7 (5)	19 (4)	0.51

Abbreviations - SD: standard deviation; RV: rhinovirus; CRP: C-reactive protein; MCPP: microbiologically confirmed pneumococcal pneumonia.

a. All characteristics are expressed as n (%); unless otherwise stated in the table.
b. *P*-values comparing RV+ and RV- cases from regression models adjusted for age in months and site of enrollment and for confounding covariates where applicable.
c. Underweight defined as weight for age <-2SD of the median age-sex specific WHO reference.
d. Premature birth defined as gestational age <37 weeks.
e. Abnormal Chest X-ray defined as radiographically confirmed end point pneumonia consolidation or any infiltrates.
f. A child was considered to be hypoxic if 1) a room air pulse-oximetry reading indicated oxygen saturation <90% at the two sites at elevation (Zambia and South Africa) or <92% at all other sites, or 2) a room air oxygen saturation.
g. Tachycardia defined as heart rate >160 beats per minute (bpm) if aged <11 months, heart rate >150 bpm if aged 12-35 months, heart rate >140 bpm if aged 36-59 months.
h. Tachypnea defined as respiratory rate >60 breaths/minute if aged <2 months, respiratory rate >50 breaths/minute if aged 2-12 months, respiration rate >40 breaths/minute if aged >12 month.
i. Fever defined as temperature >38°C.
j. Leucocytosis defined as white blood cell count >15 000 cells/uL if age <12 months, white blood cell count >13 000 cells/uL if age >12 months.
k. CRP defined as levels ≥40mg/mL which are considered to potentially indicate bacterial infection.
l. Blood culture positive for any non-contaminate bacteria.
m. MCPP defined when *S. pneumoniae* was cultured from a normally sterile body fluid - blood, pleural fluid or lung aspirate - or pleural fluid or lung aspirate was PCR *LytA* positive.

Table S4: The Demographical and Clinical Characteristics of Cases by Rhinovirus Infection Status for Each Southeast Asia Site Individually

**Characteristic, n(%)a**	**Thailand (n=221)**			**Bangladesh (n=525)**		
	**RV+ (n=40) N (%)**	**RV- (n=181) N (%)**	***P*-value[b]**	**RV+ (n=192) N (%)**	**RV- (n=333) N (%)**	***P*-value[b]**
Demographic and health:						
Mean Age, months (SD)	21.9 (15.1)	17.2 (12.9)	0.05	16.0 (11.7)	13.9 (11.8)	0.04
Female	15 (38)	73 (40)	0.71	66 (34)	125 (38)	0.42
Never breast fed	9 (23)	29 (16)	0.33	2 (1)	3 (1)	0.71
Under weight[c]	6 (15)	47 (26)	0.18	62 (32)	114 (34)	0.32
Day care attendance	7 (18)	20 (17)	0.89	1 (1)	0	0.32
Smoker in household	22 (55)	105 (58)	0.83	105 (55)	172 (52)	0.50
Premature birth[d]	7 (18)	37 (20)	0.50	9 (5)	21 (6)	0.47
Clinical Features:						
Very severe pneumonia	6 (15)	45 (25)	0.24	18 (9)	35 (11)	0.77
Chest X-ray abnormal[e]	13 (33)	85 (47)	0.11	78 (41)	141 (42)	0.60
Mechanical ventilation	6 (15)	47 (26)	0.20	15 (8)	28 (8)	0.68
Hypoxic[f]	17 (43)	81 (45)	0.81	60 (31)	80 (24)	0.15
Tachycardia[g]	32 (82)	135 (78)	0.55	182 (95)	307 (92)	0.42
Tachypnea[h]	30 (75)	70 (39)	<0.001	185 (96)	324 (97)	0.42
Wheezing	33 (83)	155 (86)	0.36	175 (91)	312 (94)	0.20
Fever[i]	0	23 (13)	0.08	1 (1)	4 (1)	0.45
Convulsions	3 (8)	39 (22)	0.06	0	1 (0)	0.740
Diarrhoea	3 (8)	5 (3)	0.13	16 (8)	27 (8)	0.80
Leucocytosis[j]	28 (70)	89 (49)	0.04	124 (69)	145 (48)	<0.001
CRP >40mg/l[k]	6 (15)	30 (17)	0.80	17 (9)	32 (10)	0.78
Blood culture positive[l]	0	7 (4)	0.40	2 (1)	3 (1)	0.96
MCPP[m]	0	0		0	0	
Hospital stay >3 days	4 (10)	76 (42)	0.01	38 (20)	70 (21)	0.93
Case fatality ratio	0	9 (5)	0.30	1 (1)	4 (1)	0.64

Abbreviations - RV: rhinovirus; SD: standard deviation; CRP: C-reactive protein; MCPP: microbiologically confirmed pneumococcal pneumonia.

a. All characteristics are expressed as n (%); unless otherwise stated in the table.
b. *P*-values comparing RV+ and RV- cases from regression models adjusted for age in month, site of enrollment and for confounding covariates where applicable; *P*-values could not be calculated for variables where both groups were zero, thus left blank.
c. Underweight defined as weight for age <-2SD of the median age-sex specific WHO reference.
d. Premature birth defined as gestational age <37 weeks.
e. Abnormal Chest X-ray defined as radiographically confirmed end point pneumonia consolidation or any infiltrates.
f. A child was considered to be hypoxic if 1) a room air pulse-oximetry reading indicated oxygen saturation <90% at the two sites at elevation (Zambia and South Africa) or <92% at all other sites, or 2) a room air oxygen saturation.
g. Tachycardia defined as heart rate >160 beats per minute (bpm) if aged <11 months, heart rate >150 bpm if aged 12-35 months, heart rate >140 bpm if aged 36-59 months.
h. Tachypnea defined as respiratory rate >60 breaths/minute if aged <2 months, respiratory rate >50 breaths/minute if aged 2-12 months, respiration rate >40 breaths/minute if aged >12 month.
i. Fever defined as temperature >38oC.
j. Leucocytosis defined as white blood cell count >15 000 cells/uL if age <12 months, white blood cell count >13 000 cells/uL if age >12 months.
k. CRP defined as levels ≥40mg/mL which are considered to potentially indicate bacterial infection.
l. Blood culture positive for any non-contaminate bacteria.

m. MCPP defined when *s. pneumoniae* was cultured from a normally sterile body fluid - blood, pleural fluid or lung aspirate - or pleural fluid or lung aspirate was PCR *LytA*.

Table S5: Rhinovirus-Positive Pneumonia Cases (N=912) and Controls (N=1056) overall, by site of enrolment and age group

	Numbers		Percent		aOR[a]	95% CI[a]	P-value[a]
	RV+ Cases	RV+ Controls	RV+ Cases	RV+ Controls			
**Overall**	912	1056	24	21	1.5	1.3-1.6	<0.001
**Mono-infections[b]**	481	656	12	13	0.9	0.8-1.0	0.136
**Mixed infections[c]**	431	400	13	9	1.4	1.2-1.6	>0.001
**<u>Age categories</u>**							
**1-5 months**	324	391	21	25	0.8	0.7-0.9	0.01
**6-11 months**	195	256	22	22	1.0	0.8-1.2	0.94
**12-59 months**	393	409	28	18	2.1	1.8-2.5	0.03
**<u>Site of enrolment</u>**							
**Kenya**	152	162	24	19	1.8	1.4-2.4	<0.001
**Gambia**	139	177	23	28	0.9	0.7-1.2	0.6
**South Africa**	182	194	23	24	1.0	0.8-1.2	0.843
**Mali**	112	143	17	19	1.2	0.8-1.6	0.326
**Zambia**	95	93	21	17	1.3	0.9-1.7	0.148
**Thailand**	40	92	18	14	1.5	1.0-2.3	0.05
**Bangladesh**	192	195	37	25	2.0	1.6-2.6	<0.001

Abbreviations - aOR: adjusted odds ratio; CI: confidence interval; RV: rhinovirus.

a. *P*-values and aOR of being a RV+ cases compared to a RV+ control from regression models adjusted for age in month, site of enrollment, prematurity, sex, breastfeeding practices, co-infecting viruses and bacteria where applicable.
b. RV was the only respiratory virus detected in the nasopharyngeal/oropharyngeal sample
c. Any viral respiratory coinfection with RSV (A and B), HMPV, AdV, InFV (A, B and C), PIV type 1-4, HCoV (OC43, NL63, 229E and HKU1).

Table S6: Demographic, Clinical and Laboratory Findings of Rhinovirus-Positive Pneumonia Cases (N=912) and Controls (N=1056), All PERCH Sites.

	Number		Percent				
	**RV+ cases**	**RV+ controls**	**RV+ cases**	**RV+ controls**	**aOR[a]**	**95%CI**	**P-value**
Demographic and health:							
Under weight[b]	277	127	30	12	3.2	2.2-4.6	<0.001
Male	539	538	59	51	1.5	1.1-1.9	0.01
Never breast fed	83	77	9	7	1.3	0.9-1.9	0.12
Smoker in household	340	392	37	37	1.1	0.9-1.3	0.55
Day Care attendance	126	163	1	16	0.95	0.7-1.4	0.80
1-12 months of age[c]	519	647	57	61	0.8	0.7-0.9	0.01
Premature birth[d]	22	17	3	2	0.8	0.6-1.1	0.19
RV epidemiology:							
RV Co-infections[e]	431	397	47	37	1.4	1.1-1.9	0.01
RV Mono-infection[f]	481	654	53	62	0.7	0.5-0.9	0.01
Respiratory viral co-infections in the NP/OP:							
RSV	121	24	13	2	4.4	2.3-8.4	<0.001
PIV	76	68	8	6	1.4	0.96-1.9	0.08
HBoV	138	138	15	13	1.2	0.95-1.6	0.12
HMPV	49	57	5	5	1.1	0.7-1.6	0.81
AdV	108	134	12	13	0.9	0.7-1.2	0.62
InFV A-C	5	9	1	1	0.6	0.2-1.9	0.42
HCoV	56	93	6	9	0.5	0.3-0.8	0.01
Bacterial infections in the NP/OP:							
B. pertussis	8	1	1	0	9.4	1.2-75.4	0.04
H. influenzae type b	23	23	3	2	1.2	0.6-2.1	0.61
S. aureus	129	135	13	14	1.1	0.9-1.5	0.38
H. influenzae	510	603	56	57	0.95	0.8-1.1	0.60
M. pneumoniae	8	4	2	1	0.8	0.4-1.6	0.50
C. pneumoniae	9	16	1	2	0.7	0.3-1.5	0.31
S. pneumoniae	661	860	72	81	0.6	0.5-0.7	<0.001
M. catarrhalis	616	836	68	79	0.5	0.4-0.7	<0.001
Any bacterial co-infection	845	1015	93	96	0.5	0.3-0.8	0.001

Abbreviations - aOR: adjusted odds ratio; CI: confidence interval; SD: standard deviation; NP/OP nasopharyngeal/oropharyngeal; RV: rhinovirus; RSV: respiratory syncytial Virus, HMPV: human metapneumovirus; AdV: adenovirus; PIV: parainfluenza type 1-4; HBoV: human bocavirus; HCoV: human coronavirus (OC43, NL63, 229E and HKU1) and InFV: influenza Virus (A, B and C).

a. *P*-values and aOR of being a RV+ cases compared to a RV+ control from regression models adjusted for age in month, site of enrollment, prematurity, sex, breastfeeding practices, co-infecting viruses and bacteria where applicable.
b. Underweight defined as weight for age <-2SD of the median age-sex specific WHO reference.
c. The mean age and standard deviation (SD) for RV cases (13.1 months; SD:12.1) and RV+ controls (13.2 months; SD:12.9; *P*=0.56).
d. Premature birth defined as gestational age <37 weeks.
e. Any viral respiratory coinfection with RSV (A and B), HMPV, AdV, InFV (A, B and C), PIV type 1-4, HCoV (OC43, NL63, 229E and HKU1).
f. RV was the only respiratory virus detected in the nasopharyngeal/oropharyngeal sample

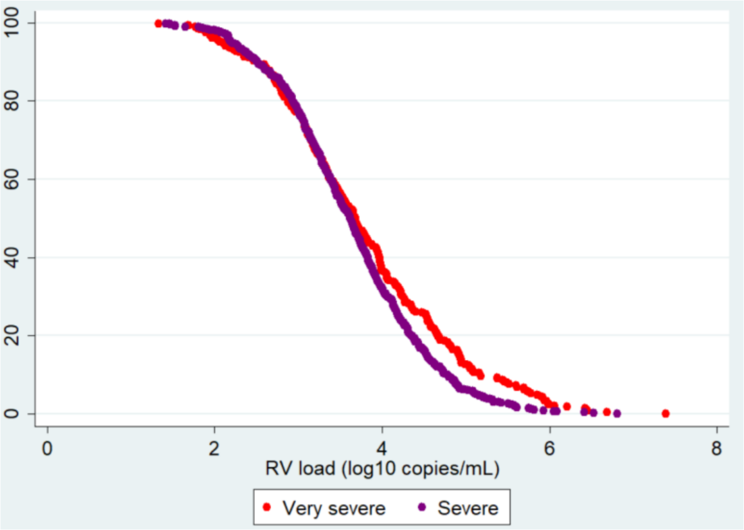

Figure S1: Reverse Cumulative Plot of RV Viral Load in the Nasopharyngeal/Oropharyngeal Sample among Very Severe and Severe Pneumonia Cases. The Viral Loads of Very Severe Cases Are Shown in Red and the Severe Cases in Purple.

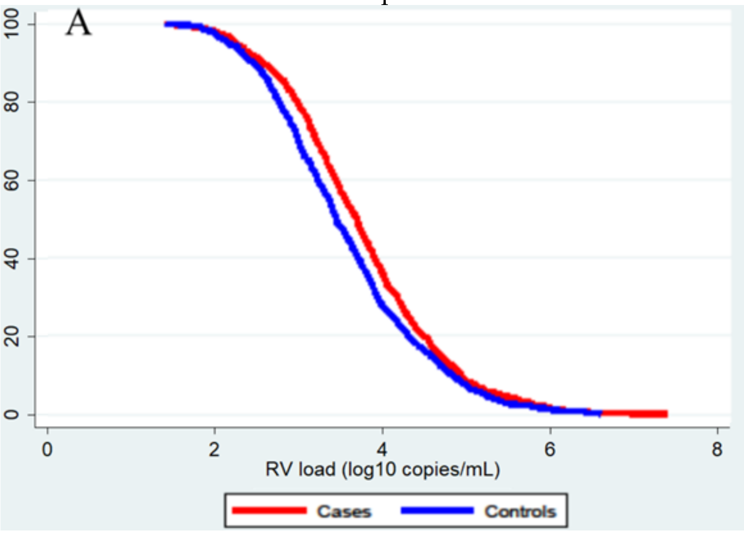

Figure S2: Reverse Cumulative Plot of RV Viral Load in the Nasopharyngeal/Oropharyngeal Sample among Cases and Controls. The Viral Loads of Cases Are Shown in Red and the Controls in Blue.
